# Spectroscopic imaging with spectral domain visible light optical coherence microscopy in Alzheimer’s disease brain samples

**DOI:** 10.1364/BOE.8.004007

**Published:** 2017-08-07

**Authors:** Antonia Lichtenegger, Danielle J. Harper, Marco Augustin, Pablo Eugui, Martina Muck, Johanna Gesperger, Christoph K. Hitzenberger, Adelheid Woehrer, Bernhard Baumann

**Affiliations:** 1 Center for Medical Physics and Biomedical Engineering, Medical University of Vienna, Waehringer Guertel 18-20, 1090 Vienna, Austria; 2 Institute of Neurology, General Hospital and Medical University of Vienna, Waehringer Guertel 18-20, 1090 Vienna, Austria

**Keywords:** (110.0110) Imaging systems, (110.4500) Optical coherence tomography, (170.0170) Medical optics and biotechnology

## Abstract

A visible light spectral domain optical coherence microscopy system was developed. A high axial resolution of 0.88 *μm* in tissue was achieved using a broad visible light spectrum (425 – 685 *nm*). Healthy human brain tissue was imaged to quantify the difference between white (WM) and grey matter (GM) in intensity and attenuation. The high axial resolution enables the investigation of amyloid-beta plaques of various sizes in human brain tissue and animal models of Alzheimer’s disease (AD). By performing a spectroscopic analysis of the OCM data, differences in the characteristics for WM, GM, and neuritic amyloid-beta plaques were found. To gain additional contrast, Congo red stained AD brain tissue was investigated. A first effort was made to investigate optically cleared mouse brain tissue to increase the penetration depth and visualize hyperscattering structures in deeper cortical regions.

## 1. Introduction

Alzheimer’s disease (AD) is the most common form of dementia and affected over 48 million people worldwide in 2015 [1]. One hallmark of AD are plaques formed out of amyloid-beta protein. Based on the presence of amyloid-beta the concept of preclinical AD has been developed [2]. Aside from research on novel diagnostic markers and therapeutic approaches, current research is focusing on unraveling the pathogenesis of AD which is still poorly understood [3].

Many imaging techniques, such as magnetic resonant imaging (MRI), positron emission tomography (PET) and computed tomography (CT), are important diagnostic tools in clinical routine and are frequently used in neuroscience research [4]. Complementing these techniques, optical coherence microscopy (OCM) could be a powerful imaging method for many in vivo and ex vivo studies, as it provides the possibility of low cost, real time, three dimensional (3D) imaging with micrometer resolution [5]. OCM is based on low-coherence interferometry and an objective lens is focusing the light onto the sample to achieve high transversal resolution [6]. The potential of OCM has already been demonstrated for diverse neuroimaging applications [7–15]. For instance, the microstructure of tumors was investigated with the help of an OCM system. In several studies, OCM provided contrast between healthy and cancerous tissues based on the difference in the backscattered and backreflected light intensity as well as on the assessment of the attenuation coefficients [7, 16, 17]. These OCM systems provided an axial resolution of 5.0 *μm*, 6.4 *μm* and 1.5 *μm*, respectively. An even higher axial resolution of 0.9 *μm* was achieved using a very broad spectrum located in the near infrared around 800 *nm* and enabled the investigation of subtle structural features of healthy and cancerous brain tissue [14]. An OCM system working in the near infrared range at 1310 *nm* was developed for deep brain tissue imaging of the cerebral cortex [8]. For some applications, OCT could even serve as an alternative to classical histology. A histological analysis requires delicate and precise slicing and staining of the tissue. This process is time consuming and prone to artifacts, such as deformations due to the tissue handling. Unlike histology, OCT offers the option of direct 3D tissue assessment. Brain tissue was investigated by polarization sensitive OCT (PS-OCT). The birefringent behavior of myelinated structures in the WM was exploited to perform tractography, i.e., orientation mapping of myelinated fibers [9,11,18]. Measurements by a PS-OCM system enabled the visualization of neuritic amyloid-beta plaques in brain tissue of human AD patients and provided information about the polarization characteristics of these pathological structures [12]. Using extended focus OCM, label free imaging of cerebral amyloid-beta plaques was demonstrated in a mouse model of AD [13].

Traditionally, light sources working in the near infrared region have been used to perform OCT, as the light can penetrate deeper into scattering tissue when working at longer wavelengths such as 1300 or 1700 *nm* [19]. Recently, imaging was also realized using visible light to perform visible light optical coherence tomography [20–24]. Major advantages of using visible light for OCT compared to near infrared light are a higher axial resolution due to a shorter central wavelength (given the same spectral bandwidth) and a stronger backscattering signal [21]. Using a very broadband light spectrum opens the door to spectroscopic imaging possibilities [21, 25–27]. Using spectroscopic visible light OCT for imaging stained tissue could be of particular interest to further increase the contrast of specific structures and compounds, similar to histological practice but without the need for sectioning [28]. Most stains and dyes, including fluorescent dyes, are designed for use under visible light and thus could be assessed by visible light OCT [29]. One drawback when working with visible light is the rather low penetration depth. In brain tissue, the penetration of visible light is limited to a couple of hundred micrometers due to strong scattering and attenuation effects [19]. One way to increase the light penetration in ex vivo tissue is to use optical clearing techniques [30]. In 2015, Murray et al. introduced an advanced clearing technique called SWITCH (system-wide control of interaction time and kinetics of chemicals) that has been successfully applied to mouse and human brain tissues [31].

In this article we present a spectral domain visible light OCM system providing sub-micrometer axial resolution with a broadband spectrometer operating at an A-scan rate of 30 *kHz*. We successfully apply it to the imaging of human brain tissue with intrinsic contrast to explore its spectroscopic imaging capabilities. In addition to OCM imaging of normal brain tissue, we showcase that visible light OCM can visualize AD-related amyloid-beta plaques in ex vivo AD brain tissue based on their inherent scattering contrast as well as by spectroscopic detection of an amyloid specific stain routinely used in neuropathology. A closer look into the distribution of these plaques in specific brain regions might help to further investigate AD and hence to gain a better understanding of the disease and to hopefully find new leads for treatment [2]. Finally, we demonstrate the first application of SWITCH clearing for visible light OCM imaging in mouse brain tissue with nearly doubled penetration depth.

## 2. Methods and materials

### 2.1. Visible light optical coherence microscopy setup

**Fig. 1:**
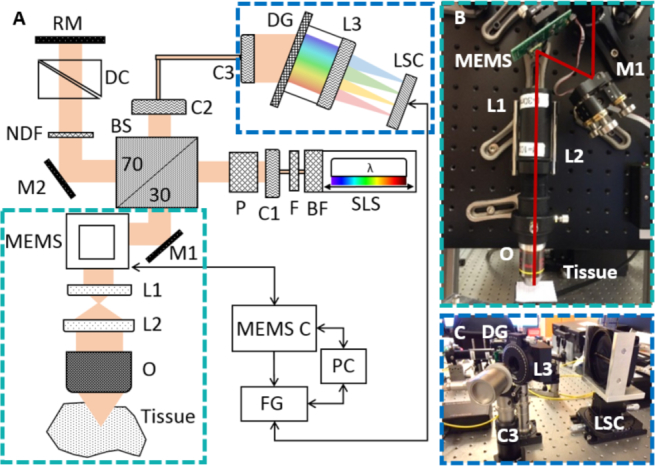
The spectral domain visible light OCM system. (a) Sketch of the system with BF (Bandpass Filter), BS (Beam Splitter), C (Collimator), DC (Dispersion Compensation), DG (Diffraction Grating), F (Filter), FG (Frame Grabber), L (Lens), LSC (Line Scan Camera), M (Mirror), MEMS (Microelectromechanical Mirror), MEMS C (MEMS Control), NDF (Neutral Density Filter), O (Objective), P (Polarizer), PC (Computer), RM (Reference Mirror), SLS (Supercontinuum Light Source). (b) Image of the sample arm. (c) Image of the spectrometer.

Imaging was performed with a visible light spectral domain OCM system [32]. A sketch of the system is shown in Fig. 1(a)

**Fig. 2:**
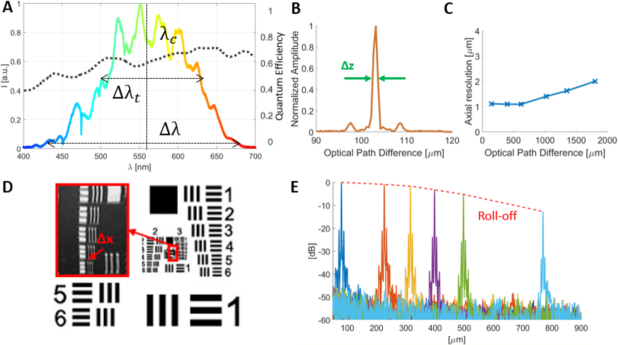
Specifications of the visible light OCM system, (a) The spectrum as measured by the spectrometer with central wavelength *λ_c_* = 555 *nm* and full-width at half maximum Δ*λ_t_* = 156 *nm* and the quantum efficiency (dotted line) of the line scan camera (data were taken from the Basler sprint user’s manual 2015 [34]). (b) Axial resolution (Δ*z* = 1.2 *μm*) measurement in the first 500 *μm*. (c) The axial resolution over the whole depth range. (d) Transversal resolution (Δ*x* = 2 *μm*) measurement with the US Air Force 1951 resolution test target. (e) Roll-off measurement at six depth positions.

. The light source was a supercontinuum laser (NKT Photonics, SuperK EXTREME EXU-6) with an emission spectrum ranging from ultraviolet (380 *nm*) to near infrared (2350 *nm*). A variable bandpass filter (NKT Photonics, SuperK VARIA) was used to crop the spectrum to the visible light range (420 – 700 *nm*). The detected spectrum, 425 – 685 *nm*, had a central wavelength of *λ_c_* = 555 *nm*, a full-width at half maximum (FWHM) of Δ*λ_t_* = 156 *nm* and a total bandwidth of Δ*λ* = 260 *nm*, see Fig. 2(a)

. The input light was attenuated after the variable bandpass filter before entering the interferometer, such that the sample arm power was 0.8 *mW*. The beam entered the system through a reflective collimator and passed a Glan-Thompson polarizer before being split into reference and sample arm by a 70:30 beamsplitter. In the reference arm, a variable neutral density filter was used to control the reference power and glass prisms (BK7, UV-Fused Silica) were inserted to compensate for dispersion effects. The sample arm, see Fig. 1(b), comprised a microelectromechanical mirror (MEMS) scanner (Mirrorcle Technologies, Inc.) to perform the raster scanning. Sawtooth functions with a fast horizontal x axis and a slow vertical y axis were applied for scanning. A telescope expanded the beam diameter to 3.6 *mm* (1*/e*^2^) to fit the aperture of the objective (Olympus UMPLFLN 10XW) with a 10× magnification to focus the beam onto the tissue. Backscattered and backreflected light from the sample and reference arm was interfered at the beam splitter and coupled into a photonic crystal fiber (NKT Photonics, LMA-5), which provides single mode transmission of almost the full visible spectrum, leading to a custom-made spectrometer, Fig. 1(c). The spectrometer included a reflective collimator, a diffraction grating with 1800 *lines/mm* and a custom made lens to focus the beam down to a CMOS line scan camera (Basler, spL8192-70km, 12-bit, pixel size 10 *μm ×* 10 *μm*) with 8192 pixels, enabling a spectral resolution of ~ 0.03 *nm*. The camera was running at a rate of 30 *kHz* and the quantum efficiency data are shown in Fig. 2(a) as a dotted line. Spectral data were collected by a frame grabber (National Instruments, NI PCIe-1473R).

Specification measurements were performed to characterize the system. An axial resolution of 1.2 *μm* in air was measured with a mirror as the sample, which corresponds to 0.88 *μm* in brain tissue assuming a group refractive index of 1.36 [33], see Fig. 2(b). The axial resolution over the whole depth range is shown in Fig. 2(c). The side lobes in the point spread function are a result of spectral modulations caused by the used optical components and the variable bandpass filter. A transversal resolution of 2 *μm* was measured by imaging a US Air Force resolution test target (Edmund Optics), Fig. 2(d). The theoretical transversal resolution was calculated as 1.8 *μm*. Resulting from the spectral resolution of ~ 0.03 *nm*, the imaging depth in air was 1.8 *mm* (1.3 *mm* in tissue). The theoretical depth of focus was 44 *μm* (NA = 0.1). Further specification measurements revealed a sensitivity of 89 *dB* close to the zero delay and a roll-off of 24 *dB/mm*, Fig. 2(e). For every OCM volume scan 500 × 500 A- and B-scans were acquired. The field of view varied from 0.25 × 0.25 *mm*^2^ to 0.5 × 0.5 *mm*^2^ for different acquisitions. One acquisition took 8.3 seconds.

### 2.2. Brain samples

#### 2.2.1. Human brains

Post mortem, formalin fixed human brain samples of one control subject and two patients diagnosed with end-stage AD (Patient 1: female, 78 years, showed additionally cerebral amyloid angiopathy and subarachnoid hemorrhage; Patient 2: female, 88 years, showed additionally complex tauopathy, argyrophilic grain disease and TDP-43 proteinopathy) were investigated. The human brain samples were provided by the Neurobiobank of the Medical University of Vienna (ethics approval number 396-2011). Specimens were obtained from patients who underwent autopsy at the Medical University of Vienna. The human control brain tissue was taken from the frontal cortex. AD brain tissue was investigated at both the frontal and temporal cortex. For spectroscopic imaging cortical pieces of 2 × 2 × 2 *mm*^3^ were cut out from the AD brain of patient 1. These samples were stained in Congo red for 120 min and cut in half, and the sectioned surface was imaged by OCM. For comparison with the gold standard histological slices were created. For distinguishing grey matter (GM) from white matter (WM) in the human control brain tissue, Klüver-Barrera staining was used, which stained myelin structures blue and GM pink. To visualize amyloid-beta plaques the AD brain tissue was stained using Congo red (Highman method). This stain specifically attaches to amyloid [35].

#### 2.2.2. Mouse brains and optical clearing

**Fig. 3:**
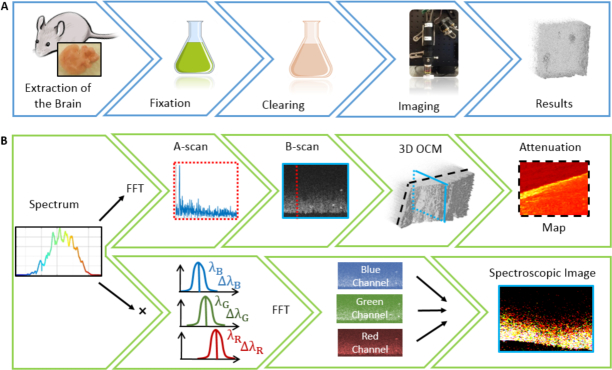
Workflow for imaging mouse brains using OCM and the post processing pipeline. (a) The first step was to extract the mouse brain, which was then fixed and clearing was performed. Imaging of the optically cleared brains was conducted and the results were analyzed. (b) Processing pipeline for attenuation en-face maps and spectroscopic OCT images. Both attenuation coefficients were calculated for 3D OCM intensity data and en-face maps were computed. For each A-scan of one B-scan the original spectrum was filtered by the chosen number of Gaussian windows to create the spectroscopic B-scans which could be combined to a spectroscopic B-scan.

Since the penetration depth in visible light OCM was limited to a couple of hundred micrometers, optical clearing of the brain tissue was performed for several samples. For this purpose the brains from healthy wild type C57BL/6 mice, 22 and 26 weeks old and one brain of an AD mouse model (APP-PS1 (amyloid precursor protein - presenilin 1 protein), 22 weeks old, female, Professor M. Jucker, Hertie Institute of Clinical Brain Research (HIH), University of Tuebingen, Germany) were used. All mice were sacrificed by cervical dislocation and the brains were carefully removed and optically cleared following the SWITCH protocol steps [31]. An overview of the steps performed for imaging the mouse brain is shown in Fig. 3(a)

. Following extraction, the mouse brain was fixed using a 4% paraformaldehyde solution for one week. It was then put into a shaker with the 4*°C* fixation-OFF solution, as described in the SWITCH protocol [31]. After two more days in the fixation-ON solution, the brain was washed two times for six hours in 4% glycerin and 4% acetamide in phosphate buffered saline and was put for one night in the inactivation solution. For the clearing step, the brain was put into the thermal clearing solution at 37°C. For a first experiment, the brain was kept in the thermal clearing solution for two days and measurements were taken after each day. In a second experiment the brain was only kept for a few hours in the thermal clearing solution and measurements were taken every 30 minutes. The AD mouse brain was cleared for 60 minutes. Animal experiments were approved by the local ethics committee and by the Austrian Federal Ministry of Science, Research and Economy under protocol BMWFW-66.009/0360-WF/V/3b/2016.

### 2.3. Data acquisition and processing

Data acquisition was performed in LabView (LabView 2015, Version 15.0, 64-bit, National Instruments) and the data were stored in a 16-bit binary format for performing further post-processing steps in Matlab (MATLAB, R2015b, MathWorks). After resampling the spectral data to k-space, background removal was performed. Numerical dispersion compensation was applied as described by Wojtkowski et al. [36] and Choi et al. [37]. By Fourier transforming these data, three dimensional OCM images were computed. En-face images were generated by calculating mean projection images at various depths within the tissue.

To further analyze the data, multiple post processing steps were performed which are summarized in a graphical overview in Fig. 3(b).

#### 2.3.1. Spectroscopic analysis

Different types of spectroscopic images were generated. Here, the general idea was to create Gaussian windows located in different wavelength regions *λ_i_* of the spectrum and to keep the axial resolution Δ*z* for each band Δ*λ_i_* constant,

$$\Delta z=\frac{2\cdot \ln(2)}{\pi }\frac{\lambda_{i}^{2}}{\Delta \lambda_{i}}.$$ (1)

A general model was established based on the given FWHM bandwidth Δ*λ_t_* of the whole spectrum and the manually chosen central wavelengths of the Gaussian windows *λ_i_* as inputs.

$$\Delta \lambda_{1}=\frac{\Delta \lambda_{t}}{1+\frac{1}{\lambda_{1}^{2}}\left(\sum_{i=2}^{N}\lambda_{i}^{2}\right)}$$ (2)

$$\Delta \lambda_{i+1}=\frac{\lambda_{i+1}^{2}}{\lambda_{i}^{2}}\Delta \lambda_{i},\;i=1\ldots N-1$$ (3)

By using equation (2) and (3), the FWHM bandwidths Δ*λ_i_* of the Gaussian windows can be calculated. The same results could be achieved by first resampling to k-space and then splitting up the spectrum in equal parts. The advantage of the approach described above is that it can be used intuitively as a toolbox only requiring the choice of select wavelength regions in order to perform spectroscopic imaging. Three different spectroscopic approaches were used with two, three and seven Gaussian windows. Table 1 gives an overview of the used total FWHM bandwidths, the central wavelength values, the calculated bandwidths and the constant axial resolutions.

**Table 1:** Parameters for spectroscopic imaging approaches.

First Approach:	Δ*λ_t_* = 156 *nm*, Δ*z* = 2.7 *μm*
	*λ*_1_ = 520 *nm*, *λ*_2_ = 560 *nm*, *λ*_3_ = 600 *nm*
	Δ*λ*_1_ = 45 *nm*, Δ*λ*_2_ = 52 *nm*, Δ*λ*_3_ = 59 *nm*

Second Approach:	Δ*λ_t_* = 55 *nm*, Δ*z* = 4.9 *μm*
	*λ*_1_ = 500 *nm*, *λ*_2_ = 600 *nm*
	Δ*λ*_1_ = 23 *nm*, Δ*λ*_2_ = 32 *nm*

Third Approach:	Δ*λ_t_* = 156 *nm*, Δ*z* = 6.2 *μm*
	*λ*_1_ = 500 *nm*, *λ*_2_ = 520 *nm*, *λ*_3_ = 540 *nm*, *λ*_4_ = 560 *nm*,
	Δ*λ*_1_ = 18 *nm*, Δ*λ*_2_ = 19 *nm*, Δ*λ*_3_ = 21 *nm*, Δ*λ*_4_ = 22 *nm*,
	*λ*_5_ = 580 *nm*, *λ*_6_ = 600 *nm*, *λ*_7_ = 620 *nm*
	Δ*λ*_5_ = 24 *nm*, Δ*λ*_6_ = 25 *nm*, Δ*λ*_7_ = 27 *nm*

For the first approach, three Gaussian windows were created such that the central wavelengths were located in the blue, green and red spectral range. By multiplying the original spectrum by these three Gaussian windows, three B-scans encoding blue, green and red light were generated after Fourier transformation. In order to achieve similar power in the three color channels, the three sub-spectra were normalized before Fourier transformation. The spectra were normalized in such a way that in the end all had the same total spectral energy (i.e. the same area under the spectral profile). Additionally in each A-scan the wavelength depended roll-off was compensated [38]. The combination of the three B-scans resulted in an RGB image, in which regions appearing white indicate equal contributions of all three spectral bands. While the chosen Gaussian windows were overlapping in the first approach, see Fig. 7(d), the idea behind the second spectroscopic image approach was to use only two Gaussian windows which were not overlapping and therefore each image was completely independent of the other (see Fig. 7(e)). The first Gaussian window was located in the green and the second in the red spectral region. Finally also a set of seven Gaussian windows covering the whole spectrum was generated, see Fig. 6(a).

#### 2.3.2. Attenuation analysis

In another processing step, the light penetration characteristics were analyzed by extracting the attenuation behavior using two different approaches. The intensity of the light amplitude *I* in a homogeneous medium follows the Lambert law of the form

$$I(z)=I_{0}\exp(-\mu_{t}z)$$ (4)

where z denotes the depth in *mm*, *μ_t_* the total attenuation coefficient in *mm*^−1^ and *I*_0_ the input intensity [39]. An exponential decay model was used to fit the attenuation coefficient for each A-scan in a certain depth beneath the surface and will henceforth be called the global attenuation coefficient. Furthermore a discrete model proposed by Vermeer et al. [39] was used to create B-scans containing the attenuation characteristics by calculating the attenuation coefficients *μ_t_*[*i*] for each pixel *i* from the intensity values *I*, the total number of pixels along an A-scan *N* and the pixel size Δ in mm by

$$\mu_{t}[i]=\frac{1}{2\Delta }\log\left(1+\frac{I[i]}{\sum_{i+1}^{N}I[i]}\right).$$ (5)

This attenuation coefficient is termed as the local attenuation. The advantage of the second method is that the attenuation can be calculated pixel wise.

### 2.4. Statistical analysis

Box and scatter plots were generated to visualize differences in the results. Two-sample t-tests in combination with Bonferroni correction were performed for statistical analysis. The significance level was defined as p < 0.05.

## 3. Results

### 3.1. Imaging of control human brain tissue

**Fig. 4:**
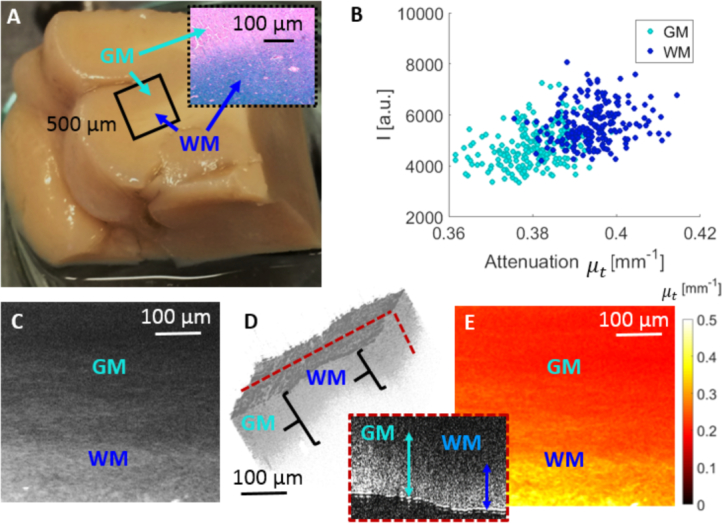
OCM imaging of healthy human brain. (a) Healthy brain tissue, where a black square indicates the scanning area and the corresponding histological image including a region of white matter (WM) and grey matter (GM). (b) Scatter plot with intensity values vs. global attenuation coefficients for GM and WM region. (c) Average projection en-face image generated over the first 100 *μm* below the surface. (d) Rendering of 3D OCM intensity image (inverted grey scale) and a B-scan showing a tissue region including GM and WM. (e) Local attenuation map generated over the first 100 *μm* below the surface.

In order to demonstrate the performance of the OCM system for ex vivo imaging of biological tissue, initial experiments were performed in formalin fixed post mortem human brain samples. Figure 4

shows results for the investigation of control brain tissue using visible light OCM. Figure 4(a) shows the scanned part of the tissue including both GM and WM as well as the corresponding histological image. To further quantify the backscattered intensity difference between GM and WM, the intensity values in a slab extending over 200 *μm* in one B-scan of WM and GM were extracted, respectively. The average intensity of the backscattered signal was higher in the region of WM compared to GM in the first 200 *μm* of the tissue. For this region the global attenuation coefficient was also extracted for each A-scan by fitting the exponential model Eq. (4) to the depth profile line. A scatter plot, consisting of 400 data points (200 for WM and 200 for GM), displaying intensity vs. global attenuation coefficients is shown in Fig. 4(b). Clusters representing GM and WM can be clearly identified. Figure 4(c) shows an average projection en-face intensity image generated over the first 100 *μm* below the surface of the brain tissue. A 3D inverted grey scale rendering of the OCM intensity data and a B-scan of a region of WM and GM is shown in Fig. 4(d). Deeper light penetration into the region of grey matter compared to white matter was observed. The local attenuation values were calculated for each pixel in the volume using Eq. (5). A local attenuation en-face map was created over the first 100 *μm* below the surface shown in Fig. 4(e).

### 3.2. Imaging of brain tissue of human AD patients

**Fig. 5:**
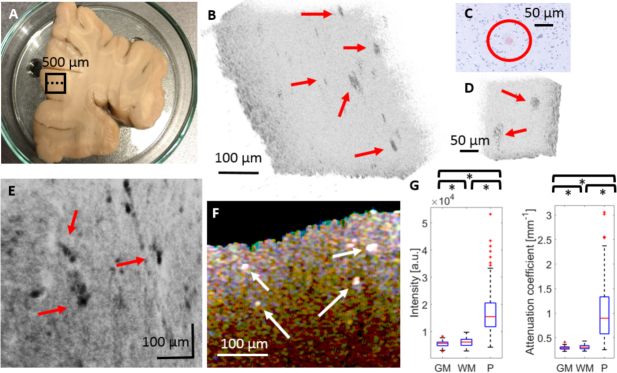
OCM imaging in the brain of a human AD patient. (a) Photograph of the AD brain tissue with the scanned area marked with a black square. (b) Rendering of 3D OCM intensity image (inverted grey scale) with amyloid-beta plaques marked with red arrows. (c) Histological example image of a neuritic plaque stained with Congo red. (d) Zoom in a plaque-rich region. (e) Mean intensity projection en-face image of AD tissue over a depth of 200 *μm* (inverted grey scale). (f) Spectroscopic B-scan of AD brain tissue using three Gaussian windows. (g) Box plots of signal intensity and local attenuation coefficients for GM, WM and plaques (P). Significant differences (p < 0.001) are indicated by asterisks.

In the next step, we investigated the capability of the system to visualize micrometer scale lesions in pathologic tissue based on their intrinsic optical properties. Cortical tissue of a human brain affected by AD was imaged using OCM, see Fig. 5(a)

. In the 3D OCM image (Fig. 5(b)), hyperscattering structures can be observed, which correspond to neuritic amyloid-beta plaques found in GM in histology. Figure 5(c) shows the histological image of a Congo red stained GM region of the corresponding tissue sample including a neuritic plaque. A zoom into a region of plaques is shown in Fig. 5(d). These structures are also clearly visible in the en-face projection image (mean intensity projection over the first 200 *μm* in the brain tissue), see Fig. 5(e).

When looking at the spectroscopic B-scan (Fig. 5(f)) generated using three Gaussian windows in the red, green and blue range, the plaques appear highly scattering in all wavelength ranges hence showing up as white in the image. The investigation showed that the plaques are visible even without additional spectroscopic analysis. Furthermore a transition from white to yellow and to red was observed due to the difference in penetration of the different wavelengths. To quantify the difference between the optical properties of GM, WM and plaques, intensity values and local attenuation coefficients were extracted from GM, WM and plaque regions, respectively. For WM and GM each 200 data points were extracted from a region of 100 *μm ×* 200 *μm* beneath the tissue surface. Likewise 200 data points were collected from various plaques. The results are visualized in two box plots in Fig. 5(g). It can be observed that the overall intensity of light backscattered from WM was higher than in GM and highest in plaques. Similar to the control brain, the attenuation coefficients were greater for WM than for GM. For the plaques, a very broad spread of attenuation coefficients can be observed. Statistically significant differences (p < 0.05) were found when comparing intensity and attenuation coefficients for WM compared to GM, WM compared to plaques and GM compared to plaques, respectively, indicated with asterisks in Fig. 5(g). To correct for multiple comparisons the Bonferroni correction was applied.

**Fig. 6:**
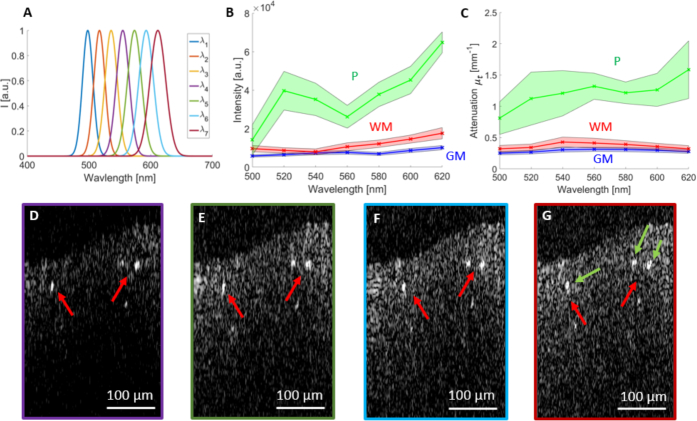
Spectroscopic evaluation of AD brain tissue. (a) The seven Gaussian windows used for the spectroscopic analysis. (b) Intensity against wavelength for WM, GM and plaques with the wavelength dependent standard deviations of the data represented by shaded bands. (c) Local attenuation coefficients against wavelength for WM, GM and plaques with the wavelength dependent standard deviations of the data represented by shaded bands. (d)-(g) B-scans of the same region as seen by different wavelength regions, (d) *λ*_4_ = 560 *nm*, (e) *λ*_5_ = 580 *nm*, (f) *λ*_6_ = 600 *nm*, (g) *λ*_7_ = 620 *nm*. Red arrows indicate the plaques and the green arrows mark the plaques where the values were extracted from.

The spectroscopic differences between WM, GM and plaques were investigated with a higher number of Gaussian windows. Seven Gaussian windows were generated using equation (2) and (3) shown in Fig. 6(a)

. B-scan images were computed for each of these windows. Out of these intensity B-scans, local attenuation coefficient images were calculated and finally average intensity and local attenuation values were extracted from a region including only WM, GM or plaques. For the analysis of the GM and WM for each wavelength region 2000 and for the plaques 200 data points were used. Figure 6(b) shows the normalized intensity values plotted against wavelength for GM, WM and plaques. In Fig. 6(c) the local attenuation coefficients are plotted against wavelength. Local attenuation is first increased and then decreases at longer wavelengths for GM and WM. Figure 6(d) and Fig. 6(g) shows the same B-scan for four different wavelength regions.

### 3.3. Imaging of stained AD human brain tissue

**Fig. 7:**
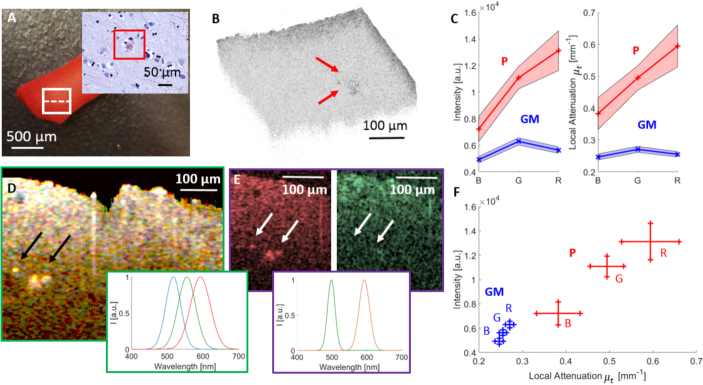
Evaluation of measurements of Congo red stained AD brain tissue. (a) Image of the Congo red stained brain tissue, where the scanning area is indicated by a white square. The insert shows a micrograph of the corresponding histology. (b) 3D OCM image including plaque regions indicated by red arrows. (c) Plots showing the intensity and the local attenuation against the three wavelength regions with the wavelength dependent standard deviations represented by shaded bands. (d) Combined spectroscopic OCM image with three Gaussian windows. Plaques are indicated by black arrows. (e) Spectroscopic OCM image computed using two Gaussian windows. (f) Intensity against attenuation plot for the three wavelength regions with the respective wavelength dependent standard deviations.

To demonstrate the potential of visible light OCM to image intact tissue processed by a standard staining protocol, AD brain tissue was stained with Congo red, see Fig. 7(a)

. In the OCM image (Fig. 7(b)), the highly scattering plaques can be distinguished from brain tissue based on the higher signal intensity. The stained AD brain tissue was analyzed by creating spectroscopic images with three and two Gaussian windows. Figure 7(c) shows the average intensity and the attenuation coefficients in GM and plaques in the red, green and blue wavelength region respectively. For averaging, a region of 100 *μm* × 100 *μm* in GM (4000 data points) and the plaque region indicated by a black arrow in Fig. 7(d), was chosen (350 data points) for each wavelength.

Different characteristics for the three color regions between GM and plaques can be observed. Obvious from the photograph of the stained AD brain tissue, (Fig. 7(a)) a stronger scattering of red light in the higher wavelength regions, can be observed. The same behavior is reflected in the results, leading to higher backscattered intensity values and stronger attenuation of higher wavelengths. Also in the stained tissue, the plaques exhibited a higher intensity and also a stronger attenuation compared to GM. The spectroscopic B-scans are shown in Fig. 7 (d) for three windows and Fig. 7(e) for two windows. In the combined spectroscopic B-scans Fig. 7(d), the plaques stand out as highly scattering features. In Fig. 7(d), the plaques appear in the red and partly in the green channel as the Gaussian windows are overlapping. It can also be observed that longer wavelengths can penetrate deeper into the tissue while the lower wavelength components of the spectrum only penetrate a few tens of micrometers deep. The difference between Fig. 7(d) and Fig. 7(e) is that in the latter only two non overlapping Gaussian windows were used as an input for filtering the broadband visible light spectrum. In Fig. 7(e) the red channel is predominantly picking up the plaque structures as an effect of Congo red staining. In Fig. 7(f) a plot of intensity vs. local attenuation coefficient is shown. Here, plaque structures and GM can easily be distinguished based on their spectral characteristics.

### 3.4. Imaging of optically cleared mouse brain tissue

**Fig. 8:**
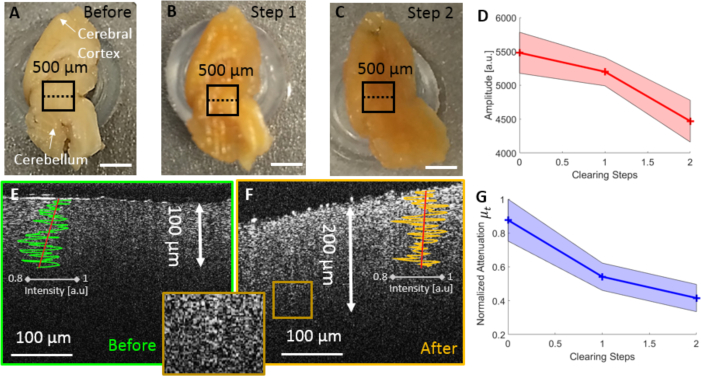
Investigating the cleared mouse brain. (a) Mouse brain before optical clearing. (b) Mouse brain after 1 day in thermal clearing solution. (c) Mouse brain after 2 days in thermal clearing solution. The black square indicates the area scanned by OCM. (d) Mean OCM amplitude extracted from a region of 100 *μm* × 100 *μm* beneath the surface versus clearing steps with the standard deviations of the data represented by shaded bands. (e) OCM B-scan structural image before clearing with an A-scan and the fitted global attenuation line. (f) OCM image after one day in Thermal Clearing Solution and zoom into a structural detail (potentially a vessel) in a deeper tissue area. (g) Mean global attenuation in a region of 100 *μm* × 100 *μm* beneath the surface versus clearing steps with the standard deviations of the data represented by shaded bands. After imaging the values were normalized with respect to the measurement before performing the optical clearing.

Optical tissue clearing was implemented in order to expand the imaging performance of visible light OCM for deep tissue regions. Figure 8

shows results of the investigation of control mouse brain tissue optically cleared using the SWITCH approach. In Fig. 8(a), an image of a formalin fixed mouse brain hemisphere is shown. Figure 8(b) shows the mouse brain after one day in thermal clearing solution. A more brownish hue of the brain and an increased transparency of the superficial tissue structures can be observed. Figure 8(c) shows the same brain after two days in the optical clearing solution with an even more brownish color and again increased transparency. OCM data sets were acquired in the prefrontal cortex of the mouse brain for each of these three steps.

One B-scan was analyzed at each of the three clearing steps and a decrease in average intensity was observed, see Fig. 8(d). For the analysis for each step 2000 data points were extracted from a region of 100 *μm* × 100 *μm* beneath the tissue surface. OCM B-scan images before clearing (Fig. 8(e)) and after one day in the thermal clearing solution (Fig. 8(f)) were acquired. Before clearing, the penetration was limited to approximately 100 *μm*, whereas after one day of optical clearing, the penetration was already increased to almost 200 *μm*. Some structural details can even be observed at a depth of 200 *μm*, see Fig 8 f). When continuing the clearing process after day one, the backscattered intensity after the surface reflection reached the level of the background such that the contrast was no longer sufficient for OCT imaging. For each step 200 data points were extracted in a region of 100 *μm* × 100 *μm* beneath the tissue surface and the global attenuation coefficients were calculated. Two example fits for A-scans before and after clearing can be seen in Fig. 8(e) and Fig. 8(f). The global attenuation coefficient was decreasing as expected, leading to a deeper penetration of the light into the tissue due to less scattering, see Fig. 8(g).

**Fig. 9:**
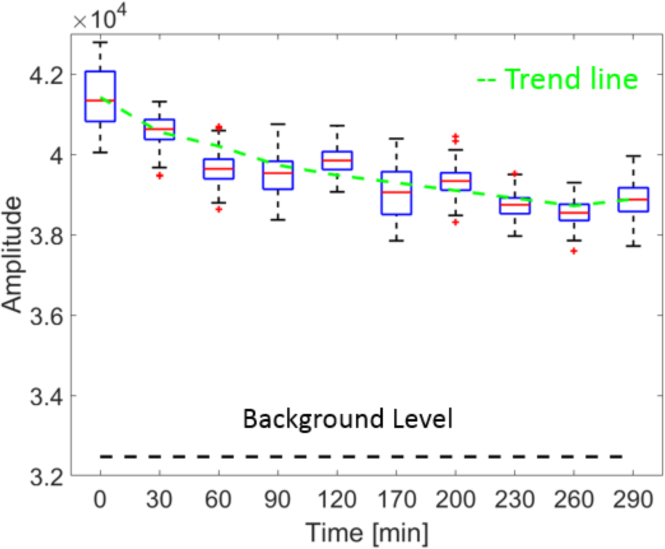
Boxplots showing the OCM signal amplitude values at various time points during the optical tissue clearing process. The trend line was computed from the mean values at each time point. The background amplitude is shown as a dashed black line for reference.

To explore the optimal clearing time, another experiment was performed. A whole mouse brain was imaged before starting the clearing process. Then the brain was put into Thermal Clearing Solution at 37°*C*. OCM measurements were taken approximately every 30 minutes at the same location over a time frame of approximately 5 hours. An area covering 200 × 100 pixels (100 *μm* (x) × 50 *μm* (z)) beneath the tissue surface was chosen to extract the mean amplitude in x direction. The time course of these values during the optical tissue clearing process is plotted in Fig. 9

. A trend of decreasing amplitude over time can be observed, as shown by the green dashed line in Fig. 9.

**Fig. 10:**
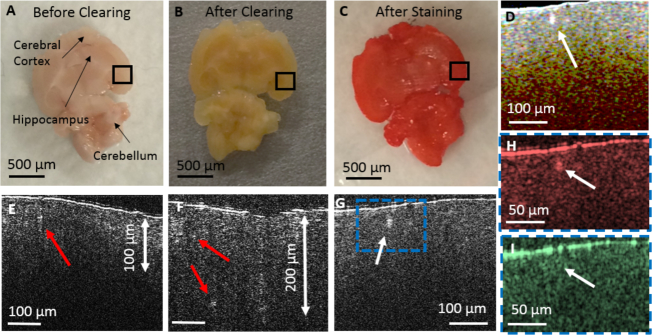
Investigating a cleared AD mouse brain. (a) Mouse brain before optical clearing, where the scanning area is indicated by a black square. (b) Mouse brain after 60 minutes of clearing. (c) Cleared and Congo red stained AD mouse brain. (d) Spectroscopic image (three Gaussian windows) of Congo red stained AD mouse brain tissue. (e) OCM B-scan of mouse brain tissue before optical clearing. (f) OCM B-scan of mouse brain tissue after performing optical clearing. (g) OCM B-scan image of Congo red stained AD mouse brain. (h) Red channel from the two Gaussian windows. (i) Green channel from the two Gaussian windows.

Finally an AD mouse brain was extracted, fixated and sliced to 1 *mm* thick coronal sections (Fig. 10(a)

) and optically cleared for 60 minutes (Fig. 10(b)). After clearing, the brain was stained for 10 minutes using Congo red (Fig. 10(c)). Imaging was performed at each step in the optical clearing and staining process. When looking at the spectroscopic image (Fig. 10(d)), the plaque structures were identified as highly scattering. OCM images reconfirmed the doubling of penetration depth (Fig. 10(e) and Fig. 10(f)) and therefore the highly scattering structures in deeper cortical areas were visible. In Fig. 10(g) a B-scan of the Congo red stained brain tissue is shown. When performing the spectroscopic analysis with two separated spectral bands, the additional structures only appeared in the red channel (Fig. 10(h)) and were not visible in the green one (Fig. 10(i)).

## 4. Discussion

In this article, a visible light spectral domain optical coherence microscopy system was used to investigate brain tissue samples. The visible light OCM system presented in this paper was based on a supercontinuum laser feasible for OCT imaging. With a bandwidth of 260 *nm* centered at 555 *nm* an ultrahigh resolution of 0.88 *μm* was achieved thereby providing access to imaging subtle structural details in healthy and pathological brain tissue such as amyloid-beta plaques. OCT based on visible light was shown to be beneficial for imaging with extremely high axial resolution as well as for performing spectroscopic measurements [20–24,26,40,41]. Ultrahigh axial resolution can be achieved by selecting a light source providing a short central wavelength and/or a very broad spectral bandwidth. In the past, visible light OCT was demonstrated for imaging subcellular components in human cells, small animals, as well as the retina and skin in humans. Thereby, axial resolutions from 0.75 *μm* to 2.0 *μm* were reported [20–24]. Owing to the rather high relative intensity noise of the supercontinuum laser, the system sensitivity was relatively low at 89 *dB* compared to the use of a standard superluminescent diode operating in the near infrared [23, 42, 43]. Similar sensitivity values were previously reported for visible light OCT systems based on supercontinuum generation [20–22, 44]. Also there is still some potential in increasing the signal in the short wavelength region, which was limited by the cutoff wavelength of 425 *nm* for the photonic crystal fiber (NKT Photonics, FD1-PM) currently used for light delivery. By using a different fiber, the spectral range could be expanded to include more of the blue spectrum, potentially even into the ultraviolet [45]. Finally the spectrum was subject to a fixed modulation pattern in the blue wavelength region, which was caused by the wavelength filter as can be observed in Fig. 2(a). A custom filter set could be used to avoid these modulations and thus to achieve a smoother spectral envelope. Despite these limitations, the OCM setup reported in this article proved considerable advances in terms of covered bandwidth, spectral resolution and imaging speed based on a custom spectrometer design. The A-scan rate of 30 *kHz* would facilely enable in vivo OCM imaging of small animals in developmental studies, thereby facilitating spectroscopic imaging of dynamic processes.

The imaging performance of the OCM system was evaluated by distinguishing grey and white matter. As expected from previous works in the near infrared [9, 10, 16], our investigations revealed that GM and WM also provided intrinsic contrast in the visible range. Significantly different penetration depths were observed in OCM images of WM and GM. WM structures are densely myelinated and therefore highly reflective, leading to strong backscattering observed at the tissue surface [8]. In contrast GM is mostly built up by cell bodies which are less scattering and hence lead to lower backscatter intensity near the brain surface [8]. Owing to the lower attenuation, light can penetrate deeper into GM compared to WM. As a result, WM and GM were easily distinguishable in OCM en-face attenuation images (Fig. 4(e)). A quantitative comparison of intensity values and attenuation coefficients revealed statistically significant differences between WM and GM (p < 0.001). The measured mean global attenuation coefficients of 0.39 (±0.06) *mm*^−1^ for WM and 0.35 (±0.03) *mm*^−1^ for GM, both calculated over the whole spectrum, were in good agreement with values reported in the literature, ranging from 0.1 to 0.9 [46–49]. However, there is a large variety in the calculated attenuation coefficients. One reason is the lack of an additional calibration step before measuring. For the future a phantom of known attenuation will be used for calibration. An example for a phantom used for calibration was shown by [50]. Furthermore the attenuation of non-fixed and formalin fixed tissue differs considerably [51]. Lastly, errors introduced by tissue distortions or incorrect surface segmentation can also affect the calculations. Recently, clusters of scatter intensity and attenuation characteristics were introduced as a sensitive method for differentiating WM and GM [52]. Consequently, a more specific differentiation between WM and GM was also observed by clustering intensity and attenuation coefficients in the visible spectrum (Fig. 4(b)). The combination of different optical properties in such clusters could enable automated differentiation and segmentation of cerebral tissue structures.

Investigating amyloid-beta plaques and in particular their accumulation behavior and structure is an important topic in neuroscience research since it is believed that amyloid-beta plaques are a biomarker for Alzheimer’s disease [2, 53, 54]. Various imaging techniques have been demonstrated for imaging cerebral amyloid-beta plaques in situ [55–58]. Since the size of amyloid-beta plaques is only in the order of a few tens of micrometers [59], high spatial resolution is required for imaging. Using molecular-targeting vectors labeled with MRI contrast agents and extremely high field strengths, it is possible to visualize single plaques using MRI [60]. Optical imaging techniques are usually less complex and more affordable. Hence a great variety of optical microscopy approaches has been demonstrated for imaging amyloid-beta plaques in the brain [58,61,62]. One drawback of many microscopy techniques is that histological tissue preparation including sectioning and staining is required prior to imaging. OCM provides rapid 3D microscopy imaging of biological tissue based on intrinsic contrast and has recently also been demonstrated for the visualization of cerebral amyloid-beta plaques using two different approaches. In a mouse model of AD, plaques were investigated by an extended-focus OCM system operating at 850 *nm* [13]. The setup featured an axicon lens in the sample arm to create a Bessel-like illumination beam for an extended focus. Using this illumination in combination with a Gaussian beam detection similar to dark-field microscopy, amyloid-beta plaques could be assessed both in vivo and in vitro [13]. Sub-micrometer resolution imaging of amyloid-beta plaques in a mouse model of AD was recently demonstrated by a visible light OCM setup which was also based on a Bessel-like illumination beam [63]. This work emphasized the need of ultrahigh resolution when visualizing amyloid-beta plaques. Especially in early stages of AD, for example to follow up plaque growth, micrometer-scale resolution may be necessary. Visible light provides this very high resolution and at the same time the possibility to perform spectroscopic imaging. In a different approach, the polarization behavior of amyloid-beta plaques in human brain samples was investigated by a PS-OCM system operating at 840 *nm* [12]. Based on their intrinsic birefringence, the three dimensional distribution of neuritic plaques was successfully visualized. In line with these earlier OCM approaches, the system presented here showcased the visualization of neuritic amyloid-beta plaques in brain samples of AD patients. Other than the three methods described before, the visible light OCM layout is relatively simple, and the setup and alignment of its optical components was straight forward. Based on the inherent hyperscattering properties of the plaques under visible light, the new approach enabled ultrahigh resolution imaging of amyloid-beta plaques in combination with spectroscopic imaging and attenuation coefficient analysis. The size and location of the plaques were also confirmed by histology (Fig. 5(c)). The imaging data in AD brain samples suggest that OCM could be utilized to collect more information about the location, distribution and plaque size variation in different areas of the brain and at different stages of the disease.

The broad spectral bandwidth opened the door to a spectroscopic image analysis of the data. Multiple spectral windows made it possible to investigate the relationship between wavelength, intensity and attenuation. For longer wavelengths, a trend towards more overall backscattering intensity was observed. Moreover the plaques, WM and GM can be distinguished based on the intensity of the backscattering signal. The spectroscopic analysis revealed a similar behavior of increasing attenuation with wavelength until 550 nm and decreasing attenuation at longer wavelengths as reported in [46, 47, 64]. This attenuation phenomenon may be explained by a combination of a continuous decrease of backscattering over wavelength and the possible absorption peak of hemoglobin at around 550 nm [47]. More investigations will be conducted to evaluate the influence of chromatic aberrations on the results. In future studies, visible light OCM may be used to automatically detect and distinguish GM, WM and plaque structures in brain tissue of AD patients or even in vivo in preclinical studies using AD models. The presented visible light OCM system may also be applied to an automated assessment of brain tumors - similar to a recent study using OCT in the near infrared [16] - based on measurements of the attenuation coefficient, or to detect lesions in other neurological diseases, such as multiple sclerosis or Parkinson’s disease.

The spectroscopic imaging capability of visible light OCM provided access to specific contrast generated by tissue staining. Staining with antibodies is a standard method in histopathologic practice and a myriad of commercially available stains and dyes have been established. Since stained tissue sections are usually examined under white light, most of the stains absorb light in the visible range and could therefore be accessible by visible light OCM. In order to demonstrate the concept of imaging stained tissue samples by visible light OCM, we performed Congo red staining of cortical samples of an AD patient, as this stain specifically attaches to amyloid [35]. By performing a spectroscopic analysis of Congo red stained tissue, the stained amyloid-beta plaques were identified based on their specific spectral contrast (Fig. 7). Congo red staining yielded a much stronger signal of the plaques in the red channel. The spectroscopic analysis revealed that the attenuation is first increased and decreased after 500 nm for grey matter. The observed chromatic attenuation may be a result of a combination of two effects: On one hand a decrease in scattering with increasing wavelength, on the other hand the absorption behavior of Congo red itself [65]. Note that the mean values for intensity and attenuation were calculated over a region of 100 *μm* × 100 *μm* of the tissue. Red light can penetrate deeper into tissue, resulting in the average intensity remaining higher for longer wavelengths. Therefore, the observed measurements seem plausible. Depending on the spectral signature of a stain, dedicated spectral windows can be tailored for the targeted detection of select structures or compounds. Also fluorescent dyes are common in histopathology and could be detected via their specific absorption band in the visible range. Furthermore, the implementation of an additional channel for detecting fluorescence would be straight forward in visible light OCM because the same light source and illumination path could be used for both modalities. By using standard dyes, the concept of visible light OCM could therefore be exploited to provide three dimensional images with molecular contrast without the need for slicing.

Imaging optically cleared brain tissue provides the possibility to increase light penetration into tissue. The disadvantage of performing optical coherence microscopy with visible light rather than with conventionally used near infrared light is the reduced penetration depth [19]. To overcome this limitation in ex vivo samples, an effort was made to increase the penetration depth by optical tissue clearing. Optical clearing was proven to increase the penetration of light in near-infrared OCT for example in skin tissue, cervical epithelium and brain tissue [66–68]. Tissue clearing is a compromise between penetration and backscattering, as the clearing process takes out the light-scattering lipid bilayers. Therefore, an additional investigation was performed in order to determine the optimal clearing time for visible light OCM imaging in murine cerebral tissue. A whole mouse brain was repeatedly imaged for several clearing steps such that signal intensity could be tracked as a function of time in the OCM images. During this longitudinal imaging process, we observed that it was very important to image a flat surface to achieve a homogeneous backscattering signal. In future investigations, a vibratome could be used to ensure a flat imaging surface [10, 18]. Investigating a cleared AD mouse brain confirmed the increase of penetration by imaging additional hyperscattering structures in deeper cortical areas. Spectroscopic analysis of Congo red stained AD mouse brain tissue demonstrated structures which were specifically stained by Congo red. In future research we are also planning to explore other clearing protocols in order to achieve the best possible image contrast while improving visible light penetration [69]. Combining optical clearing for increased light penetration, staining for specific contrast on top of the intrinsic tissue contrast, and spectroscopic visible light OCM may open a new toolbox for three dimensional deep-tissue imaging with microscopic resolution.

## 5. Conclusion

A spectral domain visible light OCM system was developed to investigate healthy and AD ex vivo brain tissue of humans and mice. GM and WM were distinguished based on the difference in backscattered and reflected intensity and attenuation coefficients. A spectroscopic analysis of the data was performed. Amyloid-beta plaques in AD brain tissue were visualized. The analysis of the results showed a statistically significant difference between plaque structures, GM and WM. A spectroscopic analysis of GM, WM and plaques was performed using two to seven spectral windows to characterize the wavelength dependence of these three structures. In order to demonstrate the potential of visible light OCM for imaging with contrast provided by stains commonly used in histopathological practice (in addition to the intrinsic contrast) Congo red stained brain tissue was imaged and the spectroscopic behavior of stained tissue was analyzed. Optical clearing increased the penetration depth and structures in deeper brain areas became visible. OCM results were in good agreement with histology. In the future more brain samples shall be investigated to get more data on the distribution and size of the plaques in various brain regions and at different stages of the disease.
